# Non-small cell lung cancer presenting with choroidal metastasis as first sign and showing good response to chemotherapy alone: a case report

**DOI:** 10.1186/1752-1947-4-185

**Published:** 2010-06-19

**Authors:** Abhishek Singh, Parul Singh, Kamal Sahni, Preety Shukla, Vikas Shukla, Nirdosh K Pant

**Affiliations:** 1 Department of Radiation and Clinical Oncology, Swami Rama Cancer Institute, UFHT Medical College, Haldwani, 263139, India; 2 Department of Ophthalmology, UFHT Medical College, Haldwani, 263139, India; 3 Department of Radiation Oncology, GSVM Medical College, Kanpur, 208002, India; 4 Department of Neurosurgery, GSVM Medical College, Kanpur, 208002, India

## Abstract

**Introduction:**

Metastatic tumors are the most common intra-ocular malignancies and choroid is by far the most common site for intra-ocular malignancies. Multiple foci are usually involved, and bilateral involvement is frequently seen. The primary sites for choroidal metastasis in decreasing order and by gender are: breast, lung, unknown primary, gastrointestinal and pancreas, skin melanoma and other rare sources in females, and lung, unknown primary, gastrointestinal and pancreas, prostate, kidney, skin melanoma and other rare sources in males. Available treatment options are external beam radiotherapy and plaque radiotherapy, while new methods like surgical resection, transpupillary thermotherapy and intravitreal chemotherapy offer promises for the future. The use of chemotherapy alone for choroidal metastases is not widely reported.

**Case presentation:**

We report the case of a 50-year-old Indian man who had a unilateral solitary lesion in his right eye. He was found to have an adenocarcinoma of the lung with choroidal metastasis as the first presenting sign. There were no findings of metastasis involving his contralateral eye. He was administered chemotherapy based on gemcitabine and carboplatin. He had significant progressive subjective and objective improvement since his first chemotherapy. His current best corrected visual acuity is 20/60 after three cycles of chemotherapy.

**Conclusions:**

Chemotherapy alone can be used as an effective mode of treatment in patients who have primary tumors that respond to chemotherapy.

## Introduction

Metastatic tumors are the most common intra-ocular malignancies, and choroid is by far the most common site for intra-ocular malignancies. Multiple foci are usually involved and bilateral involvement is frequently seen. Available treatment options are external beam radiotherapy and plaque radiotherapy. Meanwhile, newer modalities such as surgical resection, transpupillary thermotherapy and intravitreal chemotherapy offer promises for future. The use of chemotherapy alone for choroidal metastases is not widely reported.

## Case presentation

A 50-year-old Indian man presented with headache, and blurred vision in his right eye for the last three months. He had no history of seizures, vomiting or dizziness. However, he stated that he had occasional dry cough for the past four to five months.

A thorough ophthalmic and systemic examination was carried out. Ocular examination revealed his best corrected visual acuity to be counting fingers at one foot in the right eye and 20/20 in the left eye. Results of his slit lamp examination were unremarkable. His pupils were of normal size and normal reaction. His ocular movements were normal in all gazes. His intra-ocular pressure was also normal. His systemic examination showed bilaterally symmetrical chest movements. Vesicular breath sounds were audible bilaterally, but sounds on the right side were decreased as compared to the left side. Vocal fremitus and vocal resonance were decreased over the right side from the first to fourth intercostal space. No added sounds were audible. No lymph nodes were palpable clinically. A fundus picture of his right eye showed an ill-defined, yellow-white elevated lesion in choroid about three to four times the disc diameter in size, superior-temporal to the disc. A fundus picture of his left eye was normal.

**Figure 1:**
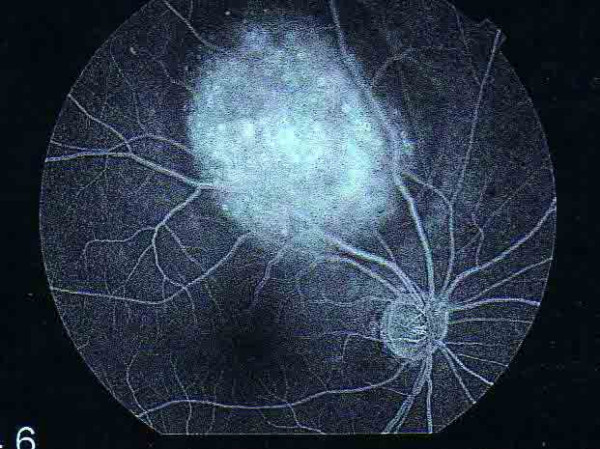
**Fundus fluorescein angiography of the involved eye**. Fundus fluorescein angiography of the right eye showing hyperfluorescence from the surface of the choroidal tumor in its late phase with the accumulation of sub-retinal fluid.

**Figure 2:**
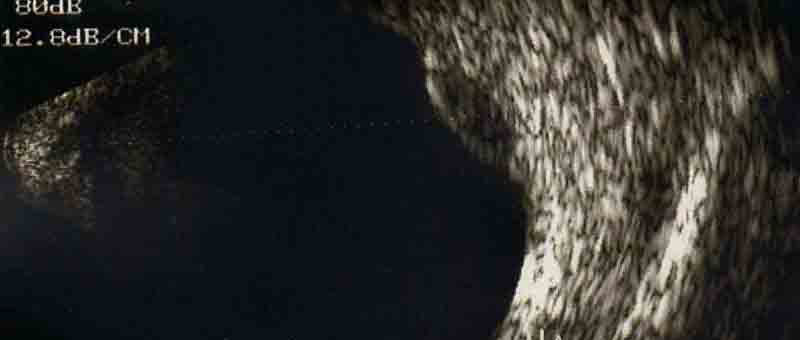
**B-scan ultrasound of the involved eye**. B-scan ultrasound showing flat-surfaced, elevated choroidal lesion with moderate internal reflectivity.

Meanwhile, fluorescein angiography of our patient's right eye revealed hyperfluorescence from the surface of his choroidal tumor. The tumor was on its late phase and it had already accumulated sub-retinal fluid (Figure 1). A B-scan ultrasound revealed a flat-surfaced, elevated choroidal lesion with moderate internal reflectivity (Figure 2). Routine systemic investigations including complete blood cell count, platelet count, bleeding time, clotting time, urine analysis, serum electrolytes, blood biochemical studies for hepatic and renal functions, as well as specific investigations like carcinoembryonic antigen, prostatic specific antigen and serum acid phosphates were all within normal limits.

**Figure 3:**
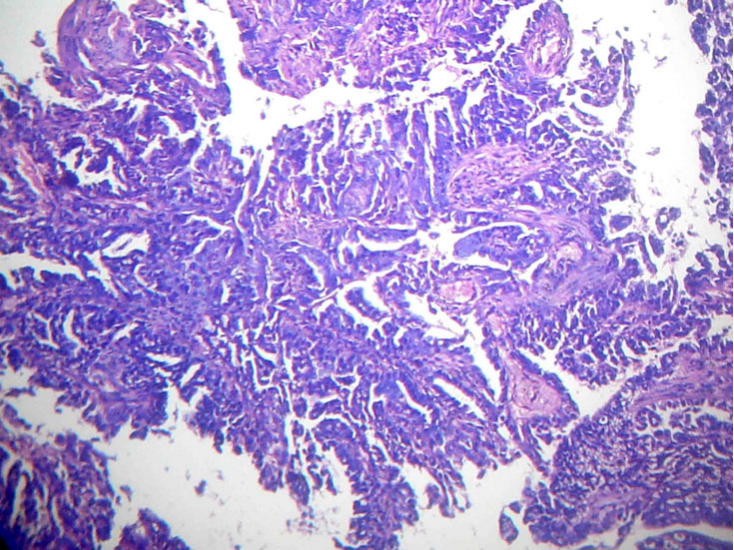
**Computed tomography-guided fine needle aspiration cytology**. Photomicrograph of fine needle aspiration cytology of the right lung lesion showing adenocarcinoma.

Results of our patient's bone scan, and upper and lower gastrointestinal series were also normal. A chest X-ray showed a homogenous opaque mass in our patient's right hilar area. His Mantoux, immunoglobulin M, and immunoglobulin G for tuberculosis tests were all negative. A computed tomography scan of our patient's thorax showed a right central bronchogenic carcinoma with ipsilateral lung having distant metastasis. Computed tomography-guided fine needle aspiration cytology from his right lung lesion was suggestive of adenocarcinoma of the lung (Figure 3). Ultrasound of his whole abdomen showed mild hepatomegaly with no focal lesions.

We prescribed six cycles of chemotherapy and the patient subsequently showed an improvement in vision. His subjective improvement after the first chemotherapy was about 50%. His best corrected visual acuity was 20/80 in the involved eye. He was administered chemotherapy based on gemcitabine and carboplatin. He had significant progressive subjective and objective improvement since his first chemotherapy. His current best corrected visual acuity is 20/30 after six cycles of chemotherapy [AU: the abstract states his best acuity after **three **cycles is **currently **20/60 - please clarify the inconsistency and/or provide more information about acuity at all treatment cycles in the body of the manuscript] in the involved eye. Recent fundoscopic examination did not show any mass.

## Discussion

Here we report a case of lung adenocarcinoma with choroidal metastasis as the first presenting sign. Our patient was administered with chemotherapy and showed a substantial improvement in vision after his first session of chemotherapy. His response to succeeding cycles has been very encouraging.

Metastatic tumors are the most common intra-ocular malignancies and choroid is the most common site for intra-ocular malignancy [1,2]. Multiple foci and bilateralism are important features of metastatic choroidal tumors. In 20% to 40% of cases, lesions are bilateral [3]. Our patient presented with a unilateral, solitary lesion along the superior temporal arcade of his right eye. Metastatic choroidal lesions are typically in the posterior pole, probably because of the relatively greater blood flow to that area [1]. Among women, the primary sites for choroidal metastasis are the breast, lung, unknown primary, gastrointestinal and pancreas, skin melanoma, and other rare sources. Among men, however, the primary sites are the lung, unknown primary, gastrointestinal and pancreas, prostate, kidney, skin melanoma, and other rare sources [1,2,4].

Shields *et al*. reported that at the time of ocular diagnosis, 66% of patients reported a history of primary cancer and 34% had no history of cancer. From 142 patients with no prior cancer, the primary site was discovered in 49% [4]. Meanwhile, Stephens and Shields reviewed 70 cases of choroidal metastasis and found that blurred vision was the presenting complaint in 80% of patients, and pain was noted in 14%, photopsia in 13%, red eye and floaters in 7% and field defects in 3% [2].

Of all patients reported to have choroidal metastasis as the presenting symptom, 58% had lung cancer and 28% had breast cancer [5]. Differential diagnosis of choroidal metastasis includes choroidal melanoma, choroidal osteoma, choroidal hemangioma, choroidal neovascularization with disciform scar, posterior scleritis and other rare lesions. Metastatic tumors usually have a creamy yellow appearance. On fluorescein angiography, these lesions are usually fluorescent in the early phases of study and become progressively hyperfluorescent in the late phases [6]. B-scan ultrasound shows an echogenic sub-retinal mass with diffuse, ill-defined borders. Overlying retinal detachment is common and sound attenuation in the lesion is usually moderate [7]. Treatment options available are external beam radiotherapy, plaque radiotherapy, and new methods like surgical resection, transpupillary thermotherapy and intravitreal chemotherapy. The doses of external beam radiotherapy required for the successful palliation of choroidal metastasis for most primary tumors is 30 grays in daily fractions of 300 centigrays. Occasionally, patients with prolonged survival are more likely to require a total dose of 45 to 50 Grays in daily fractions of 200 to 250 Grays to achieve possible long-term control [8]. In a study involving 129 patients with cancer, a recurrence rate of 7% was recorded after a median dose of 36 grays [9]. The use of chemotherapy alone for choroidal metastasis, however, is not widely reported.

Letson *et al*. described six patients with choroidal metastasis who were treated with chemotherapy and underwent regression [10]. Thus, chemotherapy alone can be used in patients with chemo-responsive primary tumor to save their vital structures from radiation. Their response to treatment can be assessed by fundoscopy, B-scan ultrasound and improvement in visual acuity.

The major determinants of survival after the diagnosis of choroidal metastasis are primary tumor type and local tumor invasion at the time of diagnosis. The median survival from lung cancer after the discovery of choroidal metastasis is reported to be 3.3 months (range 0.5 to 19 months). Our patient, described in this report, has responded well to treatment and is doing well 13 months after diagnosis.

## Conclusions

In the past, choroidal metastasis was treated with radiotherapy alone or in combination with chemotherapy. Our patient responded well after chemotherapy alone and showed marked improvement after each cycle of chemotherapy. Thus, chemotherapy alone can be a viable treatment for choroidal metastasis if the primary tumor is responsive to chemotherapy. As such, acute radiation damage and its sequelae to vital structures close to the eye can be prevented during and after radiotherapy.

## Consent

Written informed consent was obtained from our patient for publication of this case report and any accompanying images. A copy of the written consent is available for review by the Editor-in-Chief of this journal.

## Competing interests

The authors declare that they have no competing interests.

## Authors' contributions

AS was involved in the conception and design of the study, analyzed and interpreted the data, and drafted the manuscript. PS was involved in the conception, design and drafting of the manuscript. KS drafted the manuscript and revised it critically for important intellectual content. PSH was involved in the acquisition, analysis and interpretation of data and provided inputs for important intellectual content. VS interpreted the data and provided inputs for important intellectual content. NKP drafted the manuscript and revised it critically for important intellectual content. All authors read and approved the final manuscript.
